# RIP2 Is a Critical Regulator for NLRs Signaling and MHC Antigen Presentation but Not for MAPK and PI3K/Akt Pathways

**DOI:** 10.3389/fimmu.2018.00726

**Published:** 2018-04-10

**Authors:** Xiao Man Wu, Wen Qin Chen, Yi Wei Hu, Lu Cao, Pin Nie, Ming Xian Chang

**Affiliations:** ^1^State Key Laboratory of Freshwater Ecology and Biotechnology, Institute of Hydrobiology, Chinese Academy of Sciences, Wuhan, China; ^2^University of Chinese Academy of Sciences, Beijing, China; ^3^Hubei Vocational College of Bio-Technology, Wuhan, China; ^4^Key Laboratory of Aquaculture Disease Control, Ministry of Agriculture, Wuhan, China

**Keywords:** RIP2 deficiency, larval survival, transcriptome analysis, signaling pathways, NLRs signaling, MHC antigen presentation

## Abstract

RIP2 is an adaptor protein which is essential for the activation of NF-κB and NOD1- and NOD2-dependent signaling. Although NOD-RIP2 axis conservatively existed in the teleost, the function of RIP2 was only reported in zebrafish, goldfish, and rainbow trout *in vitro*. Very little is known about the role and mechanisms of piscine NOD-RIP2 axis *in vivo*. Our previous study showed the protective role of zebrafish NOD1 in larval survival through CD44a-mediated activation of PI3K-Akt signaling. In this study, we examined whether RIP2 was required for larval survival with or without pathogen infection, and determined the signaling pathways modulated by RIP2. Based on our previous report and the present study, our data demonstrated that NOD1-RIP2 axis was important for larval survival in the early ontogenesis. Similar to NOD1, RIP2 deficiency significantly affected immune system processes. The significantly enriched pathways were mainly involved in immune system, such as “Antigen processing and presentation” and “NOD-like receptor signaling pathway” and so on. Furthermore, both transcriptome analysis and qRT-PCR revealed that RIP2 was a critical regulator for expression of NLRs (NOD-like receptors) and those genes involved in MHC antigen presentation. Different from NOD1, the present study showed that NOD1, but not RIP2 deficiency significantly impaired protein levels of MAPK pathways. Although RIP2 deficiency also significantly impaired the expression of CD44a, the downstream signaling of CD44a-Lck-PI3K-Akt pathway remained unchanged. Collectively, our works highlight the similarity and discrepancy of NOD1 and RIP2 in the regulation of immune signaling pathways in the zebrafish early ontogenesis, and confirm the crucial role of RIP2 in NLRs signaling and MHC antigen presentation, but not for MAPK and PI3K/Akt pathways.

## Highlights

1. RIP2 deficiency impairs embryo hatching and larval survival
2. RIP2 deficiency impairs multiple immune signaling pathways
3. NOD1-RIP2 axis contributes to the modulation of NLRs signaling, MHC antigen presentation, and autophagy
4. Piscine MAPK pathways are activated *via* NOD1-dependent, but RIP2-independent manner
5. NOD1-mediated CD44a-Lck-PI3K-Akt pathway is independent of RIP2.

## Introduction

The receptor-interacting proteins (RIPs) are closely related to members of the interleukin-1-receptor-associated kinase (IRAK) family, and belong to a family of serine/threonine kinases. The RIP kinases function as important roles in various stimuli, such as pathogen infections, inflammation, cellular differentiation, and DNA damage (1). To date, seven different RIPs with each containing a homologous kinase domain (KD) have been described. In addition to its *N*-terminal KD, RIP1 contains a RIP homotypic interaction motif (RHIM) and *C*-terminal death domain (DD), a *C*-terminal caspase activation and recruitment domain (CARD) for RIP2, and a *C*-terminal RHIM for RIP3. RIP4 and RIP5 are characterized by the ankyrin repeats in their C-terminus (1, 2). RIP6 and RIP7 are less related in structure to the other members and contain a number of additional and diverse domain structures, such as leucine-rich repeat regions, Ras of complex proteins (Roc), and C-terminal of Roc (COR) in their N-terminus (2, 3).

RIP2, also called RIPK2, CARD3, RICK, or CARDIAK, was first described as a RIP-like kinase that had a role in NF-κB activation and apoptosis (4). NF-κB activation induced by members of tumor necrosis receptor (TNFR) family is in part mediated by TNF-receptor-associated factors (TRAF) adapter family (5, 6). RIP2 can interact with several TRAF members, such as TRAF1, TRAF2, TRAF5, and TRAF6, and induce NF-κB activation (4). In addition to NF-κB, overexpression of RIP2 also activates Jun N-terminal kinase (JNK) (7), ERK2 (8), and p38 MAPK pathways (9). The activation of ERK2, but not other activities, such as NF-κB activation, apoptosis, and JNK activation were shown to be dependent on the kinase activity of RIP2 (7, 8).

Different from other RIP kinases, RIP2 contains a C-terminal CARD which can interact with CARDs of NOD1 and NOD2, two intracellular pattern recognition receptors that sense bacterial peptidoglycans (10, 11). Subsequently, RIP2 is proven to participate in the elaboration of innate immune response to pathogens, downstream of the intracellular NOD receptors (12–14). The kinase activity of RIP2 is critical for its protein stability, and thus plays a central role in the preservation of NOD1- and NOD2-mediated innate immune responses (15). The ubiquitination of RIP2 is also a critical step in NOD1/NOD2-mediated signaling (16, 17). Many molecules that interact with RIP2 or that regulate RIP2 ubiquitination are demonstrated to be involved in the regulation of NOD1- and NOD2-mediated pathways. LIM-domain-containing protein TRIP6 interacts with RIP2 in a TNF- or IL-1-dependent manner to positively regulate NOD1 signaling (18). The E3 ubiquitin ligase ITCH directly ubiquitinates RIP2, which inhibits NOD2-induced NF-κB signaling (19). The NOD-RIP2 pathway is also targeted by caspases. Caspase-12 bound to RIP2 which led to inhibition of NOD signaling and blunting of the antimicrobial responses (20).

In mammals, much research has focused on the role of NOD-RIP2 axis in the autophagy (21) and the innate immune response against bacterial, viral, and protozoan parasite infections (22–25). NOD-RIP2 axis conservatively existed in the teleost (26), and the function of RIP2 was only reported in zebrafish, goldfish, and rainbow trout. Zebrafish RIP2 has a role in inhibiting the *Edwardsiella tarda* proliferation and SVCV (Spring Viremia of Carp Virus) replication in zebrafish embryonic fibroblast ZF4 cells (27). Goldfish RIP2 is involved in the activation of NF-κB signal pathway and the regulation of TNFα-2 and IL-1β1 production (28). In rainbow trout, inhibitor assay demonstrated that trout RIP2 was critical for expression of proinflammatory cytokines in RTH-149 cells induced by iE-DAP *via* NOD1 (29). Very little is known about the role and mechanisms of piscine NOD-RIP2 axis *in vivo*. In our previous work, we reported that NOD1 deficiency affected immune system processes including “Antigen processing and presentation” and “NOD-like receptor signaling pathway,” and that NOD1 was essential for CD44a-mediated activation of the PI3K-Akt pathway and zebrafish larval survival (30). In this report, we characterize the protective role of zebrafish RIP2 in larval survival and highlight the similarity and discrepancy of NOD1 and RIP2 in the regulation of immune system processes, MAPK and PI3K/Akt pathways in the early ontogenesis.

## Materials and Methods

### Zebrafish Care and Maintenance

The heterozygotes of *RIP2*-mutant zebrafish were obtained from the China Zebrafish Resource Center (CZRC). The homozygotic *RIP^−/−^* mutants were screened and produced in cross of heterozygous fish. Wild-type AB/TU and mutant zebrafish were raised and maintained at 28°C in the system water according to the zebrafish book. Zebrafish embryos were obtained by artificial insemination. Infected larvae for bacterial infection were always kept at 25°C.

### Zebrafish Embryo-Larval Assay

To study the effect of *RIP2* on the hatching process and embryo survival, the fertilized eggs from the WT and *RIP2^−/−^* parents were randomly divided into three experimental groups, each with 48 embryos. To exclude possible off-target effects in CRISPR-Cas9-mediated RIP2 mutation, the fertilized eggs from the WT and *RIP2^−/−^* parents microinjected with 100 ng/µl ptGFP1 or ptGFP1-RIP2 were randomly divided into two experimental groups, each with 75 embryos. The hatched larvae were recorded at 2, 3, 4, and 5 dpf to evaluate the hatching rate. The dead embryos or larvae were recorded daily until 5 dpf.

For larval survival analysis, the larvae from the WT and *RIP2^−/−^* parents at 4 dpf were randomly divided into three groups, each with 40 larvae. Dead individuals were removed and recorded daily until 7 dph (11 dpf).

### Bacterial Immersion Infection in Zebrafish Larvae

For infection of zebrafish larvae, bacteria were recovered by centrifugation, washed, resuspended in fish water. The hatched larvae (5 dpf) from WT and *RIP2^−/−^* zebrafish were exposed to 2 × 10^8^ CFU/mL *Edwardsiella piscicida* in a total volume of 5 mL. After immersion in the bacterial suspension for 6 h, zebrafish larvae were maintained in 60 mm sterile disposable petri dishes with supplemental 25 mL fresh egg water.

Exposures were performed in sextuplicate with a parallel group with 40 fish per group. The larvae in triplicate were used for survival assay. The number of surviving larvae was counted daily for 7 days. GraphPad Prism 6 was used to generate survival curves, and the log-rank test was used to test differences in survival between the WT and *RIP2^−/−^* zebrafish infected with *E. piscicida*. The other larvae in triplicate were used for measuring bacterial burden. 10 larvae per group at 1 and 2 dpi were rinsed and lysed in 500 µl of PBS *via* a glass homogenizer. Serial dilutions of the homogenates were plated onto TSB agar and CFU were enumerated after 20 h of incubation at 28°C.

### cDNA Library Construction and Illumina Deep Sequencing

Total RNA was isolated from the WT and *RIP2^−/−^* zebrafish at 7 dpf using the TRIzol^®^ Reagent (Invitrogen). cDNA libraries for whole transcriptome analysis were generated and sequenced according to the methods from our previous report (30). To identify DEGs between the WT and *RIP2^−/−^* zebrafish, the expression levels were measured by using numbers of fragments per kilobase of transcript per million fragments sequenced (FPKM). The raw sequences were deposited at NCBI Sequence Read Archive (Accession No. SRP095651).

### RIP2 Overexpression

In order to determine the effect of RIP2 overexpression on those differentially expressed genes (DEGs) involved in MHC antigen processing and presentation, and NACHT-containing proteins, 100 ng/µl ptGFP1, or ptGFP1-RIP2 were microinjected into one- or two-cell stage zebrafish embryos. At 48 h post-microinjection, 50–60 embryos or larvae per group were collected and used for RNA extraction.

### Quantitative Real-Time PCR

Quantitative real-time PCR (qRT-PCR) was performed on a BIO-RAD CFX96 Real-Time System under the following conditions: 3 min at 95°C, followed by 50 cycles of 15 s at 94°C, 15 s at 52–60°C, and 30 s at 72°C. All reactions were performed in triplicate in a 96-well plate and the mean value recorded. DEGs involved in MHC antigen processing and presentation and NACHT-containing proteins were used for validation. Those DEGs involved in MHC antigen processing and presentation, include PA28 (ENSDARG00000033144), HSP70 (ENSDARG00000021924), β2M (ENSDARG00000053136), CD74a (ENSDARG00000009087), tapasin (ENSDARG00000045011), tapasin-like (ENSDARG00000076483), ctssb.2 (ENSDARG00000013771), MHC class I (ENSDARG00000076734), mhc2a (ENSDARG00000031745), mhc2β-like (ENSDARG00000074510), and mhc2dab (ENSDARG00000079105). Those DEGs for NACHT-containing proteins, include ENSDARG00000088142, ENSDARG00000095200, ENSDARG00000068621, ENSDARG00000087532, ENSDARG00000090901, ENSDARG00000091499, ENSDARG00000089306, ENSDARG00000089179, ENSDARG00000079456, ENSDARG00000076819, ENSDARG00000093532, Danio_rerio_newGene_6897, Danio_rerio_newGene_1336, ENSDARG00000068841, and ENSDARG00000068431, which were selected for the suitable amplification primers. The housekeeping gene GAPDH was used for normalizing cDNA amounts. The primers specific for the gene of interest were listed in Table 1.

**Table 1:** Primer information.

Name	Sequence	Size	Application
PA28F	AAAGAAGAAAGCTCCCAAGT	372 bp	Quantitative real-time PCR
PA28R	TCAAGCACAATCACCCTAAT		
HSP70F	CAACAACCTGCTGGGCAAA	90 bp	
HSP70R	GCGTCGATGTCGAAGGTCA		
β2MF	GGGAAAGTCTCCACTCCGAAAG	217 bp	
β2MR	GGGTGAAGGCAACGCTCT		
CD74aF	AGAAGCAGCACATCAACG	144 bp	
CD74aR	AAGTGGGGTCAGAGTAATCC		
tapasinF	CTGGGTCAGGGCAGTGTAGTGGGT	154 bp	
tapasinR	AGGCTCGTCGTGTTCCTCCGTCAT		
tapasin-like F	AAAAGTGAAGGCAAAGGAGT	398 bp	
tapasin-like R	AGGCCAGGCTGTAGATAGAA		
ctssb.2F	CCACCCGCCCTCAGTTTATCTT	167 bp	
ctssb.2R	GTAGCCACCATCTCCAAATCCAG		
MHC class IF	TCAAGGAGGACCAGATAGA	242 bp	
MHC class IR	AAGGGTGAAGTGAATAAAG		
mhc2αF	AGCCACAGACATTAGGGCATACAA	391 bp	
mhc2αR	CAGAACCAGACCCACTCCACAGAA		
mhc2β-like F	TGGCTGAGAAATGGTGAAGT	195 bp	
mhc2β-like R	AGACGCAGGACGAGATGAA		
mhc2dabF	CTCTGTGGGGAAGTTTGTG	133 bp	
mhc2dabR	CCAGATCCGAGCATTATGTC		
ENSDARG00000088142F	CATCAGGAGGAAGGGAAGAGG	276 bp	
ENSDARG00000088142R	CCAGAATAAATCCTAAACCCAGCA		
ENSDARG00000095200F	CCAGGAGATTCAGGAGTCAAG	147 bp	
ENSDARG00000095200R	GTCTGTTTTCAAAATTCTAGTGT		
ENSDARG00000068621F	AAAGTCATTTGAAGGCACA	223 bp	
ENSDARG00000068621R	CAGAAGAAACCGCAGGAA		
ENSDARG00000087532F	CTGGACACTGTTACTGGGAGGCT	166 bp	
ENSDARG00000087532R	TGCTTGGCAATCAATCTGTTCTT		
ENSDARG00000090901F	GAGGACAAAAAAACAACATCAGTC	259 bp	
ENSDARG00000090901R	CTCACAGCGGGCACCAGTCT		
ENSDARG00000091499F	ACCTCTTTCTGCGTTTCCTGCTT	258 bp	
ENSDARG00000091499R	GCTTCTCCTCCGAGTGTTTGTCT		
ENSDARG00000089306F	GCTCTATTGGCACGATGTTA	285 bp	
ENSDARG00000089306R	CAGACGATTGTTGCGTAGGT		
ENSDARG00000089179F	GGCTCTGTTTCCTCACTC	377 bp	
ENSDARG00000089179R	CACTCCTATTCTCCTGCT		
ENSDARG00000079456F	TTTTCTTCTGGGCCTTTC	308 bp	
ENSDARG00000079456R	AATCCTCCATCTCCTGCT		
ENSDARG00000076819F	ACTGGGGTTGAGTAATTG	468 bp	
ENSDARG00000076819R	TTTGTGGAGTTTGATAGA		
ENSDARG00000093532F	GACCAACATCAAACATCAGA	342 bp	
ENSDARG00000093532R	TTAGACCACAAACTCGGCTT		
Danio_rerio_newGene_6897F	AAAACTACTTCGCTCTTCCCTAA	198 bp	
Danio_rerio_newGene_6897R	TACAGACTCCTGGATTTCTTGAT		
Danio_rerio_newGene_1336F	CTGGCAATGAGGCTTATCAGGAAA	138 bp	
Danio_rerio_newGene_1336R	TCAGGACAACAACAGGAGCAAACA		
ENSDARG00000068841F	GAATGGACATCTGGACCTTTACC	408 bp	
ENSDARG00000068841R	CTTCTGGAGGCTTTGACGACTG		
ENSDARG00000068431F	TTTCTTGAGCGGGAATACAGT	109 bp	
ENSDARG00000068431R	ATGAGGGATGGAAGTTTGGAC		

### Western Blot Analysis

For detecting the protein expression involved in MAPK/ERK pathways, autophagy and PI3K-Akt pathway, 60–250 larvae at 7 dpf from WT, *NOD1-1IS^−/−^* and *RIP2^−/−^* zebrafish were lysed in 200–800 μl cold RIPA buffer containing phosphatase inhibitor (Prod #78420) and protease inhibitor using ultrasonication. Primary antibodies used were phospho-p4442 MAPK (Cell Signaling #9101), p4442 MAPK (Cell Signaling #9102), p38 MAPK (Novus, H6-NB500-138), Atg5 (Novus, H6-NB110-53818), p62 (Sigma, P0067), LC3b (Sigma, L7543), phospho-Akt (Cell Signaling #4060S), Akt (Cell Signaling #9272), phospho-GSK-3β (Cell Signaling #9323), GSK-3β (Cell Signaling #9315), phospho-S6 ribosomal protein (Cell Signaling #2215), and S6 ribosomal protein (Cell Signaling #2217) at a dilution of 1:1,000. Mouse monoclonal anti-GAPDH (proteitech, 60004-1-Ig) was used throughout as a loading control. Secondary antibodies were diluted 1:5,000 including Pierce goat anti-rabbit IgG and goat anti-mouse IgG (Prod #31460 and #31430). The bands were detected using Pierce ECL Western Blotting Substrate (Prod #32106) and ECLWestern blot system (LAS-4000mini, Fuji, Japan) according to the manufacturer’s instructions. Densitometer analysis was performed using Quantity One software (BioRad).

### Statistical Analysis

Expression data by qRT-PCR are presented as means and standard error of mean (SEM). Two-tailed Student’s *t*-test or ANOVA were used to compare means and SEM between groups. All data are representative of two or three biologic replications. The level of significance is shown as follows: *p < 0.05, **p < 0.01. Significance testing in the cumulative survival analysis used Log-rank test.

## Results

### RIP2 Deficiency Impairs Embryo Hatching and Larval Survival

Our previous report revealed that NOD1 deficiency in zebrafish impaired embryo hatching and larvae survival (30). Since that RIP2 is a pivotal adaptor protein of NOD1-dependent signaling, and that the constitutive expression of RIP2 exists throughout zebrafish early development (26), we have interest to know whether RIP2 has similar effect. We first employed the Cas9/gRNA system to generate RIP2 knockout zebrafish. A gRNA with 20-bp “target sequence” (GGACCTGCACTACATCAGCA) was designed, and microinjected together with Cas9 mRNA into one-cell embryos of zebrafish. After crossing the F1 heterozygotes and selfing the dominant F2 heterozygotes, homozygotic *RIP2^−/−^* mutants were screened out by primer pairs and sequencing (Figure S1 in Supplementary Material).

**Figure 1:**
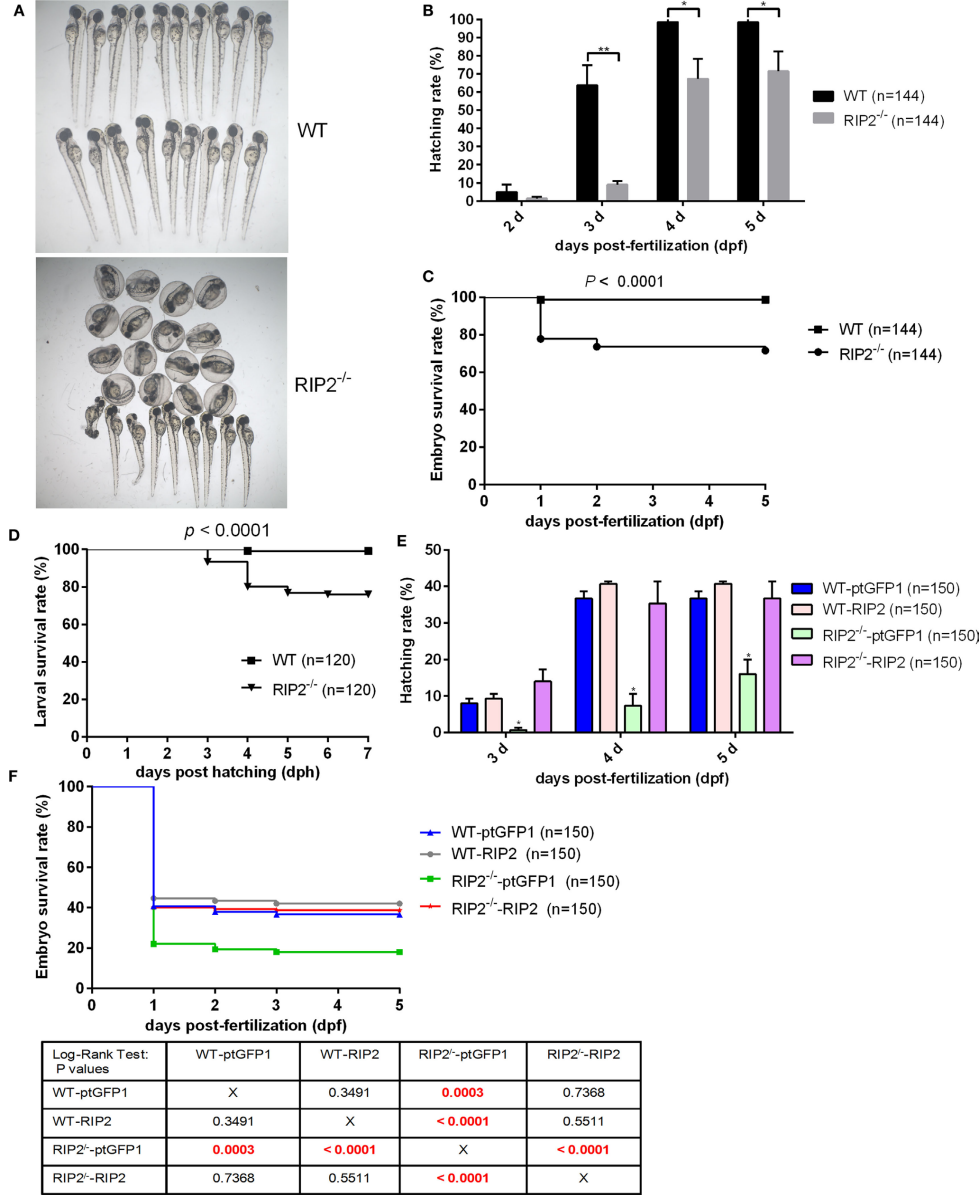
RIP2 is critical for embryo hatching and larvae survival. **(A)** The images of embryos and larvae from WT and *RIP2^−/−^* zebrafish at 3 days post-fertilization (dpf). **(B)** The hatching rate of WT and *RIP2^−/−^* zebrafish at 2, 3, 4, and 5 dpf (*n* = 3, each with 48 embryos). Data represent the mean ± the SEM and were tested for statistical significance using two-tailed Student’s *t*-test. **p < 0.01, *p < 0.05. **(C)** The embryo survival curves of WT and *RIP2^−/−^* zebrafish (*n* = 3, each with 48 embryos). **(D)** The larval survival curves of WT and *RIP2^−/−^* zebrafish (*n* = 3, each with 40 larvae). The survival curves were compared statistically significant difference using the Log-Rank Test. **(E)** The hatching rate of WT and *RIP2^−/−^* zebrafish microinjected with ptGFP1 or ptGFP1-RIP2 at 3, 4, and 5 dpf (*n* = 2, each with 75 embryos). Data represent the mean ± the SEM, and were tested for statistical significance using two-tailed Student’s *t*-test. *p < 0.05. **(F)** The embryo survival curves of WT and *RIP2^−/−^* zebrafish microinjected with ptGFP1 or ptGFP1-RIP2 (*n* = 2, each with 75 embryos). The survival curves were compared statistically significant difference using the Log-Rank Test. The *P*-values lower than 0.001 were in red.

We then evaluate the effect of RIP2 in zebrafish hatching process and larvae survival. At 2 days post-fertilization (dpf), only 4.86% of WT zebrafish embryos hatched out of the chorion, and no statistical difference existed between the WT and *RIP2^−/−^* zebrafish. At 3 dpf, about 64% of the WT zebrafish embryos hatched out of the chorion, whereas *RIP2^−/−^* zebrafish embryos hatched at a significantly decreased rate of 9.02% (Figures 1A,B). At 4 dpf, all the WT zebrafish embryos hatched out of the chorion with a 98.61% hatching rate, whereas the hatching rate of *RIP2^−/−^* zebrafish embryos only increased to 67.36%. At 5 dpf, the hatching rate of *RIP2^−/−^* zebrafish embryos increased to 71.52% (Figure 1B). Furthermore, RIP2 deficiency significantly impacts the embryo survival rate, and the deaths occurred mainly within the first day after fertilization (Figure 1C). The hatched larvae from WT and *RIP2^−/−^* zebrafish were used for survival analysis. Compared to WT zebrafish, the survival of *RIP2^−/−^* larvae was significantly decreased at 3 days post-hatching (dph), and greatly reduced at 4 dph (Figure 1D). Taken together, these results show that RIP2 deficiency in zebrafish impairs embryo hatching and larvae survival.

To exclude possible off-target effects in CRISPR-Cas9-mediated RIP2 mutation, we next evaluated the effect of RIP2 overexpression on the hatching process in the WT and RIP2 knockout zebrafish. At 3 and 4 dpf, about 8 and 36.7% of WT zebrafish embryos microinjected with ptGFP1 control plasmid hatched out of the chorion, whereas *RIP2^−/−^* zebrafish embryos microinjected with ptGFP1 hatched at a significantly decreased rate of 0.67% at 3 dpf and 7.3% at 4 dpf. Due to the high constitutive expression of RIP2 in the WT zebrafish embryos (26), RIP2 overexpression in the WT zebrafish embryos cannot significantly increase the hatching rate. However, the hatching rate of *RIP2^−/−^* zebrafish embryos can be rescued by RIP2 overexpression (14% at 3 dpf and 35.3% at 4 dpf, Figure 1E). Furthermore, the deaths in the WT and *RIP2^−/−^* zebrafish microinjected with ptGFP1 or RIP2 occurred mainly within the first day after fertilization, which was similar to WT and *RIP2* zebrafish without microinjection. The embryo survival rate of RIP2 knockout zebrafish can also be rescued by RIP2 overexpression (Figure 1F).

### RIP2 Deficiency Affects Immune System Processes in the Early Ontogenesis

**Figure 2:**
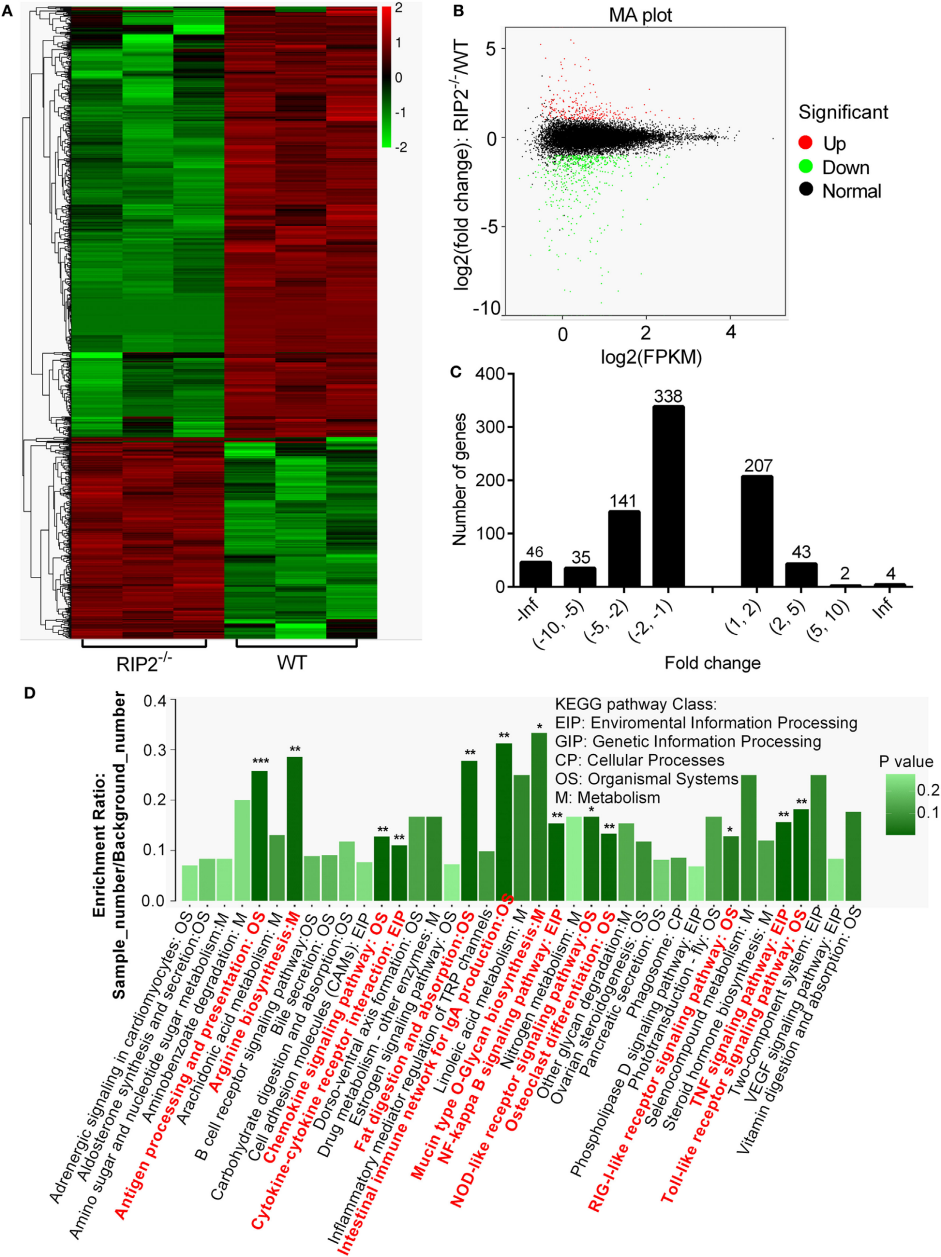
RNAseq profiling in WT vs *RIP2^−/−^* zebrafish larvae. **(A)** Heatmap analysis for transcriptomic data from the WT and *RIP2^−/−^* zebrafish larvae collected at 7 days post-fertilization. **(B)** The expression levels of differentially expressed genes (DEGs). Red spots represent DEGs for upregulation, green spots for downregulation, and black spots for unchanged genes in the *RIP2^−/−^* zebrafish larvae. **(C)** The distribution of DEGs. **(D)** The KEGG pathways regulated by RIP2. The significantly enriched pathways are in red. ***p < 0.001, **p < 0.01, and *p < 0.05.

To investigate the signaling pathways regulated by RIP2 that was involved in larval survival, transcriptome sequencing was performed using zebrafish larvae from WT and *RIP2^−/−^* mutants collected at 7 dpf (3 dph), when the survival rate of WT and *RIP2^−/−^* larvae started to diverge significantly. In total, 816 DEGs were obtained when *p*-value ≦ 0.05 and |logFC|≧1 were set as the cut-off limits (Figures 2A,B). Among them, 560 downregulated genes and 256 upregulated genes were identified (Figure 2C).

Based on NR annotations, the KEGG classification system was used to classify the possible functions of DEGs. The significantly enriched pathways were mainly involved in immune system, which included “Antigen processing and presentation,” “Toll-like receptor signaling pathway,” “TNF signaling pathway,” “NF-kappa B signaling pathway,” “Intestinal immune network for IgA production,” “Chemokine signaling pathway,” “Cytokine-cytokine receptor interaction,” “NOD-like receptor signaling pathway,” and “RIG-I-like receptor signaling pathway” with a *p*-value ≦ 0.05. In addition, these pathways including “Fat digestion and absorption,” “Osteoclast differentiation,” “Arginine biosynthesis,” and “Mucin type O-Glycan biosynthesis” were also significantly enriched (Figure 2D; Table 2).

**Table 2:** Enrichment analysis of the KEGG pathways for differentially expressed genes in WT vs *RIP2^−/−^* zebrafish larvae.

#Term	Id	Sample number	Background number	*P*-value
Antigen processing and presentation	ko04612	11	31	0.000284167
Toll-like receptor signaling pathway	ko04620	10	55	0.000599
TNF signaling pathway	ko04668	10	64	0.001674
NF-kappa B signaling pathway	ko04064	10	65	0.001856
Intestinal immune network for IgA production	ko04672	5	16	0.002014
Chemokine signaling pathway	ko04062	12	94	0.002832
Fat digestion and absorption	ko04975	5	18	0.003091
Osteoclast differentiation	ko04380	10	75	0.004696
Cytokine–cytokine receptor interaction	ko04060	13	118	0.006048
Arginine biosynthesis	ko00220	4	14	0.007467
NOD-like receptor signaling pathway	ko04621	5	30	0.019168
RIG-I-like receptor signaling pathway	ko04622	5	39	0.04625
Mucin-type O-Glycan biosynthesis	ko00512	2	6	0.047142

**Figure 3:**
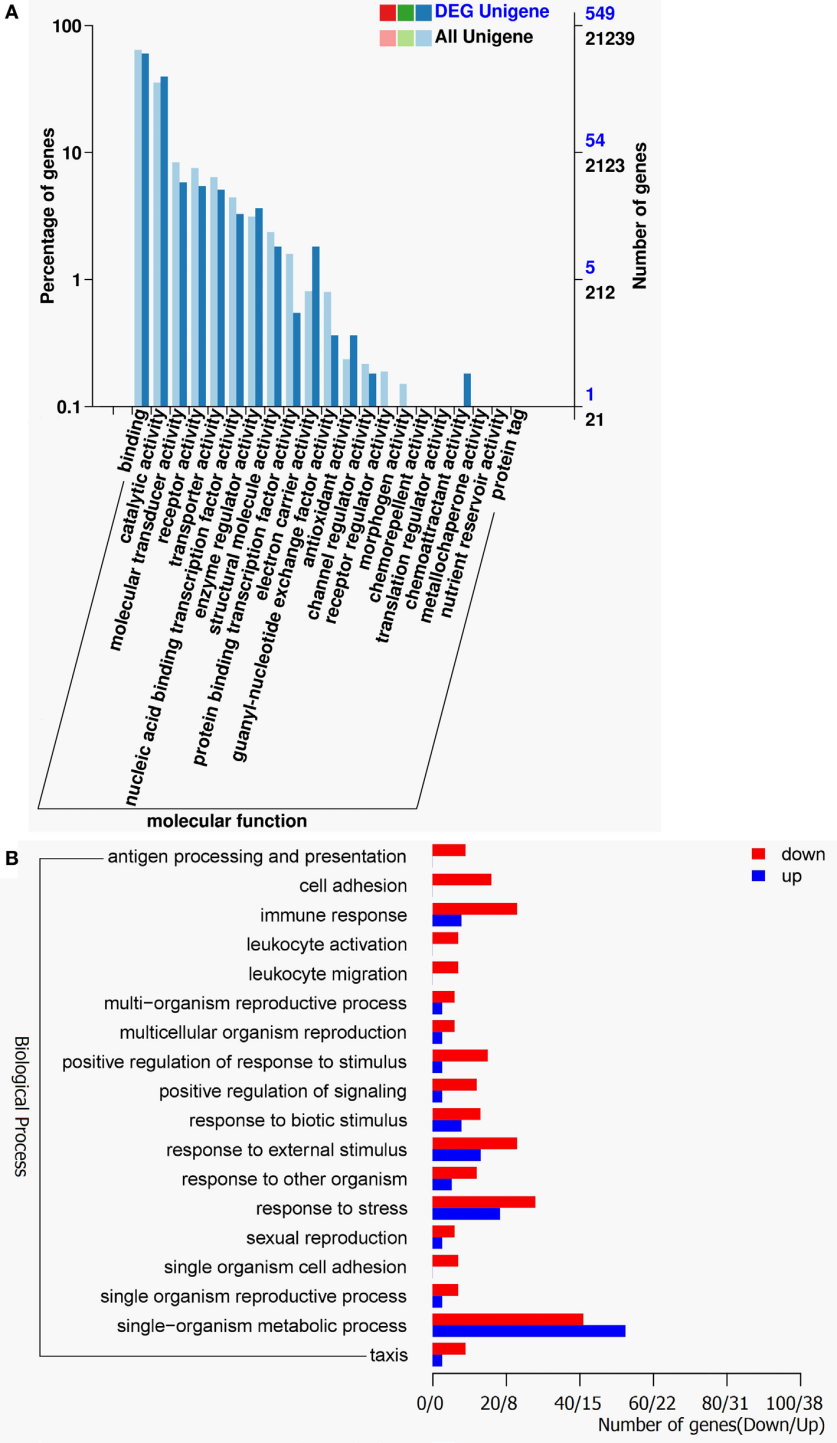
Gene ontology (GO) functional annotation. **(A)** GO functional annotation according to molecular function. In all 21,239 Unigenes and 549 DEG Unigenes were assigned to at least one GO term and were grouped into molecular function. Right *y*-axis represents number of unigenes in a category. Left *y*-axis indicates percentage of a specific category of unigenes in each main category. **(B)** GO functional annotation according to biological process. Red columns represent differentially expressed genes for downregulation and blue columns for upregulation.

The gene ontology (GO) classification system was also used to classify the possible functions of DEGs. A total of 548 genes (67.16%) were successfully assigned to at least one GO term annotation. For the molecular function category, the top two largest categories were “binding” and “catalytic activity” (Figure 3A). According to biological process, the top two largest categories were “single-organism metabolic process” and “response to stress” (Figure 3B). And, more remarkable, most genes involved in these biological process, including “antigen processing and presentation,” “cell adhesion,” “immune response,” “leukocyte activation,” “leukocyte migration,” “positive regulation of response to stimulus,” and “response to stress” were downregulated (Figure 3B). These results show that RIP2 deficiency significantly affect immune system processes in the early ontogenesis.

### RIP2 Is a Critical Regulator for MHC Antigen Presentation in the Early Ontogenesis

**Figure 4:**
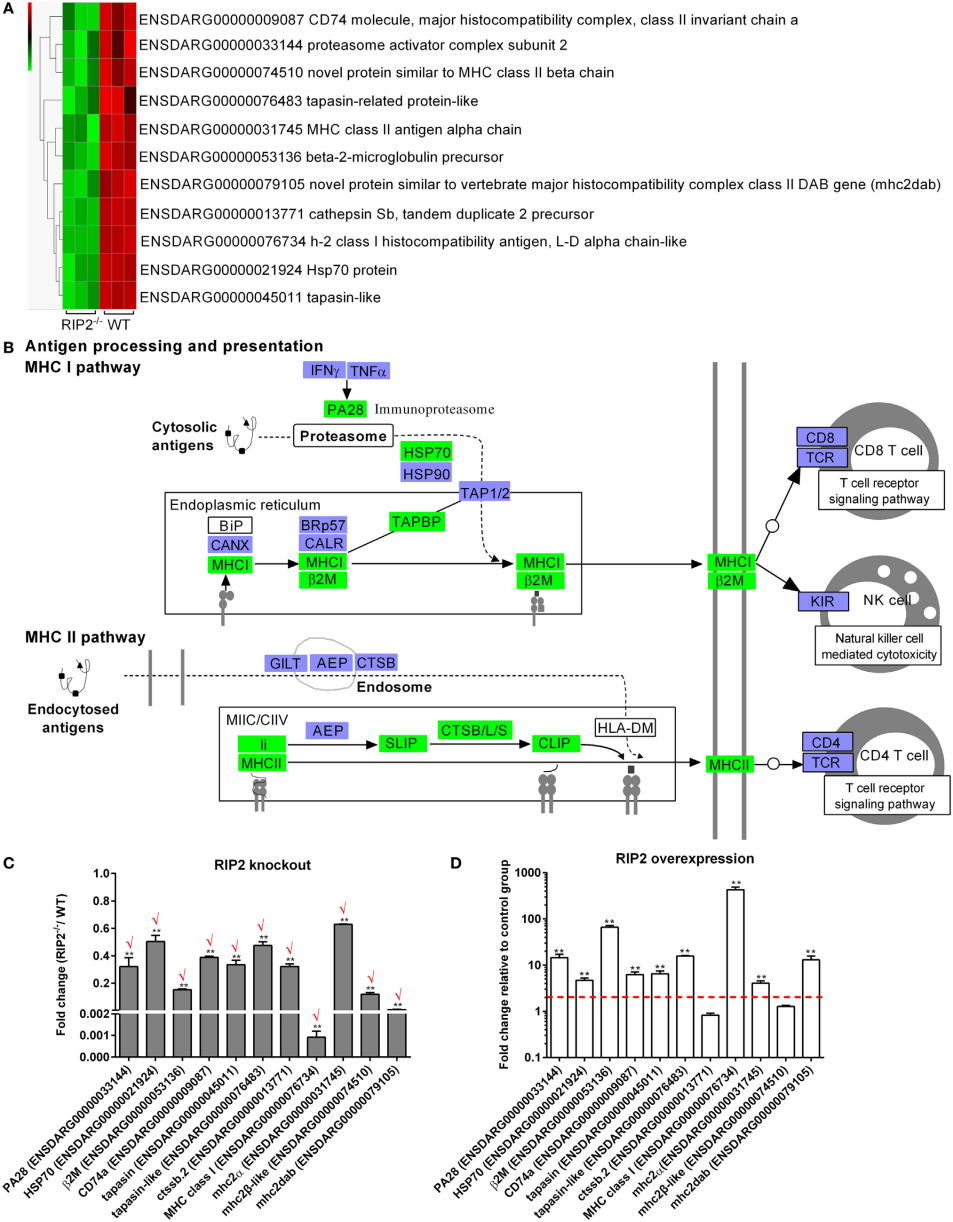
RIP2 is a critical regulator for MHC antigen presentation. **(A)** The gene cluster for “MHC antigen presentation.” At the top of this panel, a color key denotes the gradient scale of gene expression from low (green) to high (red) degrees. **(B)** The KEGG pathway for “MHC antigen presentation” regulated by RIP2. Those genes in green were significantly downregulated by RIP2. **(C)** The expression changes of those genes involved in MHC class I and II pathways in the RIP2 knockout zebrafish compared with WT. 30–50 larvae from WT and *RIP2^−/−^* zebrafish were collected at 7 days post-fertilization and used for quantitative real-time PCR (qRT-PCR). Data represent the mean ± the SEM, and were tested for statistical significance using two-tailed Student’s *t*-test. **p < 0.01. Those genes with the same expression tendency between RNA-seq and qRT-PCR are indicated by “√” in red. **(D)** The expression changes of those genes involved in MHC class I and II pathways in the RIP2-overexpressed zebrafish compared with that microinjected with empty plasmid. 50–60 embryos or larvae from ptGFP1-microinjected and RIP2-microinjected zebrafish were collected at 48 h post microinjection and used for qRT-PCR. Data represent the mean ± the SEM, and were tested for statistical significance using two-tailed Student’s *t*-test. **p < 0.01.

Comparative transcriptome analysis showed that a total of 11 genes involved in “Antigen processing and presentation” pathway, which include CD74 (ENSDARG00000009087), proteasome activator complex subunit 2 (PA28, ENSDARG00000033144), novel protein similar to MHC class II beta chain (ENSDARG00000074510), tapasin-related protein-like (ENSDARG00000076483), MHC class II antigen alpha chain (ENSDARG00000031745), beta-2-microglobulin precursor (ENSDARG00000053136), novel protein similar to vertebrate major histocompatibility complex class II DAB gene (mhc2dab, ENSDARG00000079105), cathepsin Sb, tandem duplicate 2 precursor (ENSDARG00000013771), h-2 class I histocompatibility antigen, l-d alpha chain-like (ENSDARG00000076734), Hsp70 protein (ENSDARG00000021924), and tapasin-like (ENSDARG00000045011) were differentially downregulated by RIP2 deficiency (Figure 4A). These DEGs were involved in both MHC I and II pathways (Figure 4B).

To further corroborate the correlation between RIP2 and MHC antigen presentation, we probed the expression of these identified 11 DEGs by qRT-PCR in the RIP2-deficient or RIP2-overexpressed zebrafish larvae. The results showed that RIP2 deficiency indeed impaired the expression of 11 genes, which presented 100% consistency between RNA-seq and qRT-PCR (Figure 4C). Conversely, RIP2 overexpression significantly induced the expression of PA28, HSP70, β2M, CD74a, tapasin, tapasin-like, MHC class I, mhc2α, and mhc2dab (Figure 4D). All these results show that RIP2 is a critical regulator for MHC antigen presentation.

### RIP2 Is a Critical Regulator for NLRs Signaling in the Early Ontogenesis

**Figure 5:**
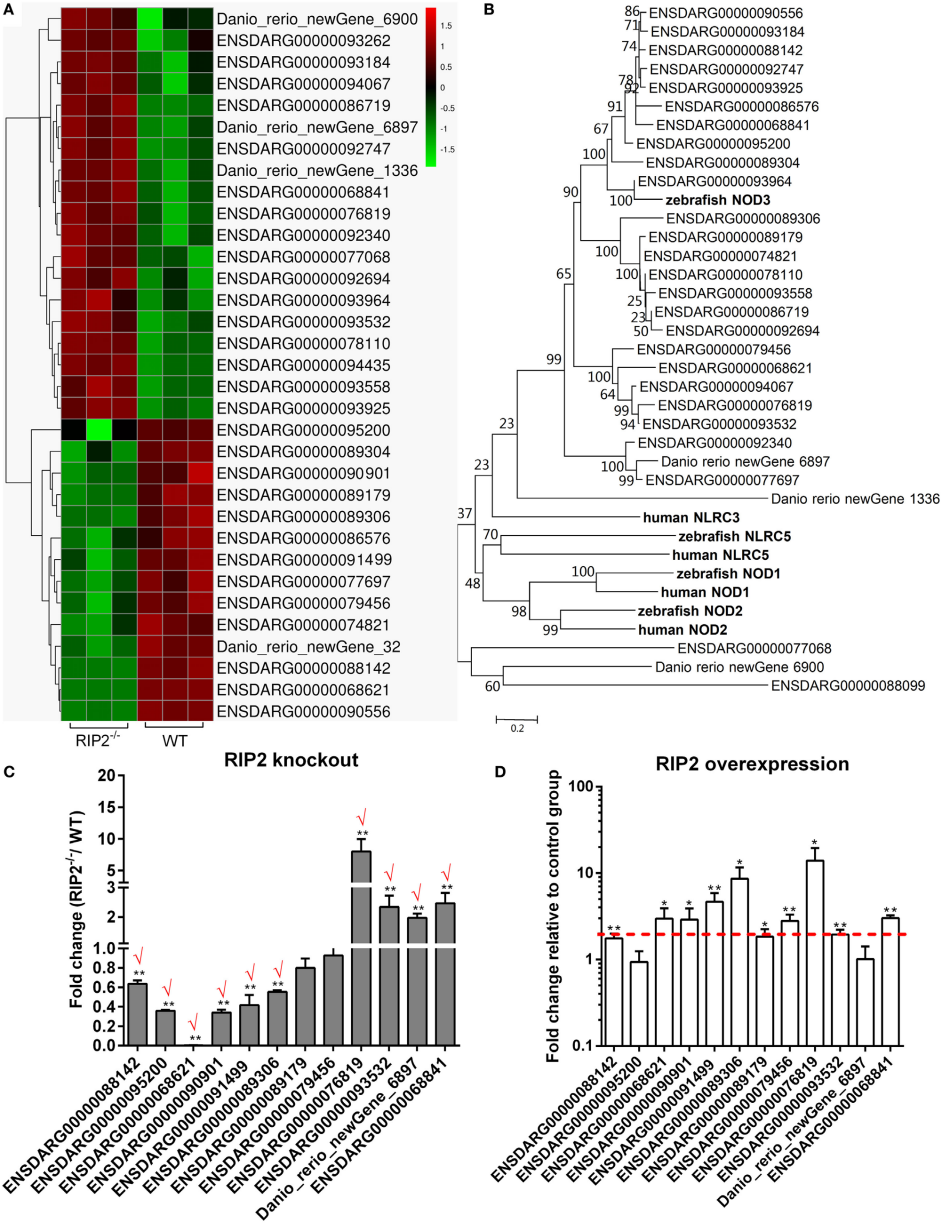
RIP2 is a critical regulator for NLRs signaling. **(A)** The gene cluster for “NOD-like receptors.” At the top of this panel, a color key denotes the gradient scale of gene expression from low (green) to high (red) degrees. **(B)** Phylogenetic tree of NOD-like receptors. Phylogenetic relationships were based on amino acid alignments. Bootstrap values based on 10,000 replicates are indicated on each branch. The evolutionary history was inferred using the neighbor joining method. All positions containing gaps and missing data were eliminated from the dataset (pairwise deletion). Accession numbers of other NLR sequences from NCBI databases are as follows: zebrafish NOD2, NP_001314973; zebrafish NOD3, XP_003201049; zebrafish NLRC5, XP_003200494; human NLRC3, ACP40993; human NLRC5, NP_115582; human NOD1, AAD28350; and human NOD2, AAG33677. **(C)** The expression changes of NLRs in the RIP2 knockout zebrafish compared with WT. 30–50 larvae from WT and *RIP2^−/−^* zebrafish were collected at 7 days post-fertilization and used for quantitative real-time PCR (qRT-PCR). Data represent the mean ± the SEM, and were tested for statistical significance using two-tailed Student’s *t*-test. **p < 0.01. Those genes with the same expression tendency between RNA-seq and qRT-PCR are indicated by “√” in red. **(D)** The expression changes of NLRs in the RIP2-overexpressed zebrafish compared with that transfected with empty plasmid. 30–50 embryos or larvae from ptGFP1-transfected and RIP2-transfected zebrafish were collected at 48 h post transfection and used for qRT-PCR. Data represent the mean ± the SEM, and were tested for statistical significance using two-tailed Student’s *t*-test. **p < 0.01.

Comparative transcriptome analysis showed that 33 NLRs containing NACHT and LRR domains were differentially regulated by RIP2 (Figure 5A). The phylogenetic analysis suggested that 10 NLRs regulated by RIP2 were the homologs of NOD3 (Figure 5B). Since RIP2 is known to be the critical adaptor protein of NOD1 and NOD2, we are interested to know whether RIP2 has any effect on the other NLRs except for NOD1 and NOD2. Twelve NLR genes were selected and used for qRT-PCR validation. Except for ENSDARG00000089179 and ENSDARG00000079456, the expression of other 10 NLRs was in agreement with their transcript abundance changes determined by RNA-seq, with 6 NLRs downregulated and 4 NLRs upregulated (Figure 5C). The correlation between RIP2 and NLRs were further validated by qRT-PCR in RIP2-overexpressed zebrafish larvae. The results showed that the expressions of 10 NLRs were increased by RIP2 overexpression (Figure 5D). It is obvious that these NLRs, including ENSDARG00000088142, ENSDARG00000068621, ENSDARG00000090901, ENSDARG00000091499, and ENSDARG00000089306 were indeed positively related with RIP2 (Figures 5C,D).

### RIP2 Is Not Essential for MAPK Pathways in the Early Ontogenesis

**Figure 6:**
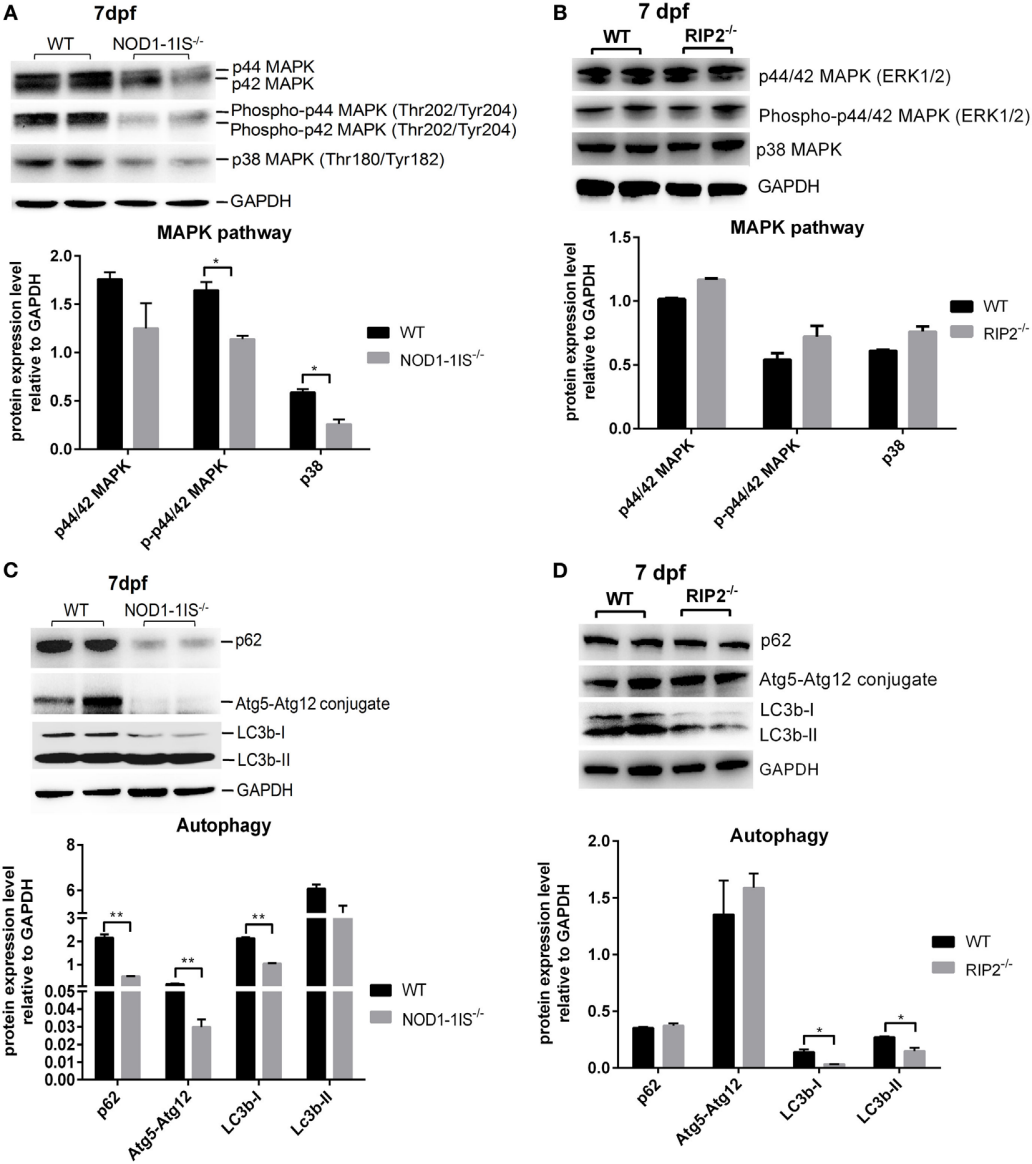
RIP2 is not essential for MAPK pathways in the early ontogenesis. **(A)** Immunoblot analysis of phospho-p44/42 MAPK, p44/42 MAPK, and p38 MAPK in larvae homogenate from WT and *NOD1-1IS^−/−^* zebrafish at 7 days post-fertilization (dpf). **(B)** Immunoblot analysis of phospho-p44/42 MAPK, p44/42 MAPK, and p38 MAPK in larvae homogenate from WT and *RIP2^−/−^* zebrafish at 7 dpf. **(C)** Immunoblot analysis of Atg5, p62, and LC3b in larvae homogenate from WT and *NOD1-1IS^−/−^* zebrafish at 7 dpf. **(D)** Immunoblot analysis of Atg5, p62, and LC3b in larvae homogenate from WT and *RIP2^−/−^* zebrafish at 7 dpf. Western blotting results were quantified using Quantity One software. Data represent the average of two independent experiments. *p < 0.05, **p < 0.01.

Previous studies showed that mammalian NOD1 and RIP2 were involved in the activation of NF-κB and MAPK pathways (4, 7–9, 31). In teleost, our previous report revealed that NOD1 deficiency did not significantly affect NF-κB and MAPK pathways at the transcriptional level (30). The present results from comparative transcriptome analysis demonstrated that RIP2 deficiency significantly affected NF-kappa B signaling pathway, but not MAPK pathway. To confirm the unchanged activities of MAPK, we performed Western blotting for MAPK target genes by using lysates from wild-type, *NOD1-1IS^−/−^* and *RIP2^−/−^* zebrafish larvae at 7 dpf. We evaluated changes in the protein expression of p44/p42 MAPK (ERK1/2), phospho-p44/42 MAPK, and p38 MAPK, all of which have been shown to play a role in the MAPK pathways, and also have commercial antibodies to be applicable for zebrafish. The impaired protein levels of phospho-p44/42 MAPK and p38 MAPK were observed in *NOD1-1IS^−/−^* zebrafish (Figure 6A). However, RIP2 deficiency has no effect on the protein expression of p44/p42 MAPK, phospho-p44/42 MAPK, and p38 MAPK (Figure 6B).

During vertebrate development, autophagy plays an essential and conserved role (32). Previous study showed that NOD1 can promote RIP2-dependent autophagy and inflammatory signaling on early endosomes in response to bacterial infection (21). To determine whether NOD1-RIP2 axis contributed to autophagy in the early ontogenesis of lower vertebrate, we performed Western blotting for autophagy machinery Atg5, autophagy adaptor protein p62/SQSTM1, and autophagy-related marker LC3 by using lysates from wild-type, *NOD1-1IS^−/−^* and *RIP2^−/−^* zebrafish larvae at 7 dpf. The impaired protein levels of p62, Atg5-Atg12 conjugate, and LC3b-I were observed in *NOD1-1IS^−/−^* zebrafish larvae (Figure 6C). Although LC3b was decreased in *RIP2^−/−^* zebrafish larvae, the expression of p62 and Atg5-Atg12 conjugate in *RIP2^−/−^* zebrafish larvae were similar to that in the WT larvae (Figure 6D), which suggested that NOD1 and RIP2-directed autophagy modulation through different mechanisms.

### RIP2 Is Not Essential for NOD1/CD44a-Mediated Activation of the PI3K-Akt Signaling in the Early Ontogenesis

**Figure 7:**
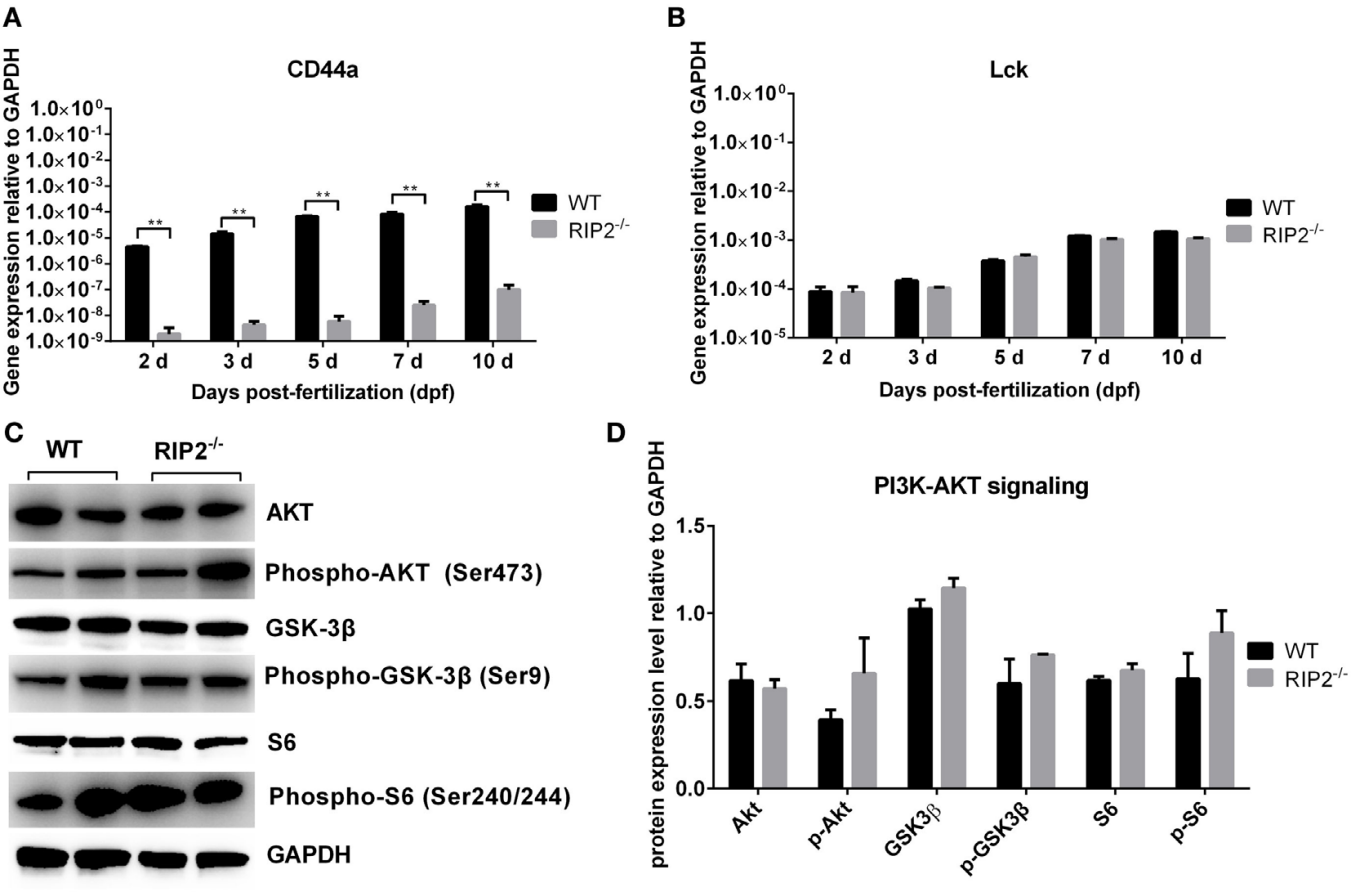
RIP2 is not essential for NOD1/CD44a-mediated activation of the PI3K-Akt signaling in the early ontogenesis. **(A)** The expression of CD44a in the WT and *RIP2^−/−^* zebrafish. **(B)** The expression of Lck in the WT and *RIP2^−/−^* zebrafish. For **(A,B)**, 30–50 embryos or larvae from WT and *RIP2^−/−^* zebrafish were collected at 2, 3, 5, 7, and 10 days post-fertilization (dpf), and used for qPCR. Data represent the mean ± the SEM. **p < 0.01. **(C)** Immunoblot analysis of Akt, phospho-Akt, GSK3β, phospho-GSK3β, S6, phospho-S6 in larvae homogenate from WT and *RIP2^−/−^* zebrafish at 7 dpf. Western blotting results were quantified using Quantity One software. **(D)** Data represent the average of two independent experiments. *p < 0.05, **p < 0.01.

Our previous report revealed that overexpression of CD44a in NOD1-deficient zebrafish can restore the impaired expression of PI3K-Akt pathway and improve larval survival, which suggested that CD44a was critical for NOD1-mediated regulation of PI3K-Akt (30). Since RIP2 was an important adaptor protein for NOD1 signaling, we also evaluated whether RIP2 was required for CD44a-Lck-Akt signaling. In *RIP2^−/−^* zebrafish larvae, mRNA transcription of CD44a was decreased two to three logs (Figure 7A), but remain unchanged for Lck (Figure 7B), a tyrosine kinase which mediated CD44 signaling (33, 34). Additionally, the levels of phosphorylated and total proteins of Akt, GSK-3β, and S6 were unchanged in *RIP2^−/−^* zebrafish larvae (Figures 7C,D). These results indicate that the downstream signaling of CD44a-Lck-Akt pathway is independent of RIP2.

### RIP2 Is Important for the Immune Control of *E. piscicida* Infection *In Vivo* in the Early Ontogenesis

**Figure 8:**
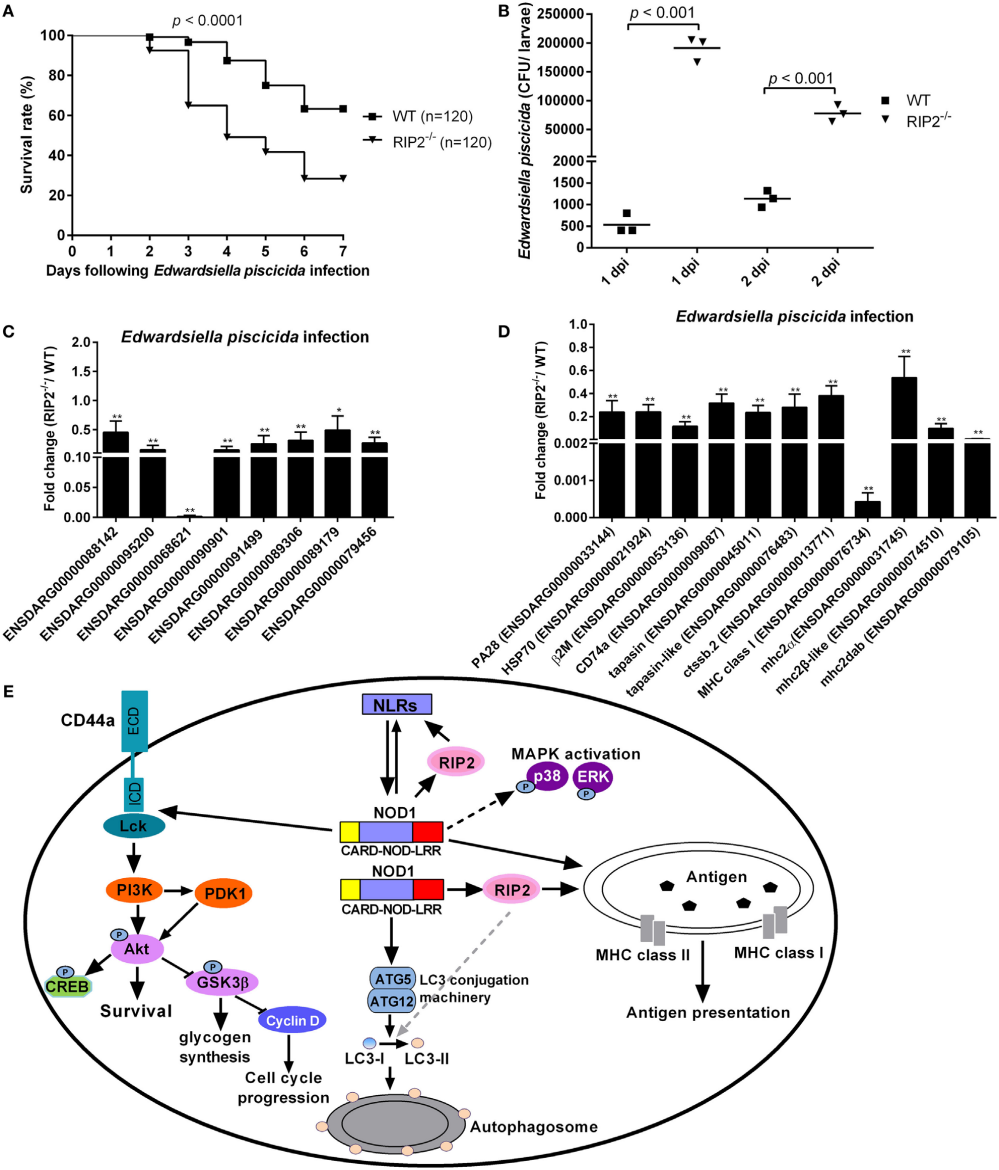
RIP2 is critical for *Edwardsiella piscicida* infection. **(A)** Survival of WT and *RIP2^−/−^* zebrafish exposed to *E. piscicida* by static immersion. Zebrafish larvae at 5 days post-fertilization (dpf) were exposed to 2 × 10^8^ CFU/mL *E. piscicida* for 6 h, and monitored for 7 days. **(B)** Bacterial loads in the WT and *RIP2^−/−^* larvae at 1 and 2 dpi. Larvae from WT and *RIP2^−/−^* zebrafish were harvested, homogenized, and plated onto TSB agar for enumeration of CFU. **(C)** The effect of RIP2 deficiency on the expression of NLRs during *E. piscicida* infection. **(D)** The effect of RIP2 deficiency on the expression of those genes involved in MHC antigen presentation during *E. piscicida* infection. For **(C,D)**, the hatched larvae (5 dpf) from WT and *RIP2^−/−^* zebrafish were exposed to 2 × 10^8^ CFU/mL *E. piscicida* in a total volume of 5 mL. After immersion in the bacterial suspension for 6 h, zebrafish larvae were maintained in 60 mm sterile disposable petri dishes with supplemental 25 mL fresh egg water. Larvae from WT and *RIP2^−/−^* zebrafish were harvested at 48 hpi and used for qPCR. Data represent the mean ± the SEM. *p < 0.05, **p < 0.01. **(E)** Proposed model illustrating the intracellular signaling pathways modulated by NOD1-RIP2 axis during zebrafish larval development at homeostasis.

To evaluate the role of RIP2 in the innate response to *E. piscicida* infection *in vivo*, WT and *RIP2^−/−^* zebrafish at 5 dpf were exposed to *E. piscicida* by static immersion. We found that RIP2 deficiency significantly decreased the survival of zebrafish larvae after infection with *E. piscicida* (Figure 8A). To examine whether differential mortality in WT and *RIP2^−/−^* zebrafish was associated with bacterial dissemination, bacterial load in whole fish was determined at 1 and 2 days post-infection (dpi). Compared with WT zebrafish larvae, significantly higher bacteria were detected in *RIP2^−/−^* zebrafish larvae at 1 and 2 dpi (Figure 8B). Thus, RIP2 protects against *E. piscicida* infection which is correlated with the extent of bacterial dissemination.

### RIP2 Deficiency Impairs the Expression of NLRs and Those Genes Involved in MHC Antigen Presentation During *E. piscicida* Infection

To determine whether RIP2 plays any role in the NLRs signaling and MHC antigen presentation in response to *E. piscicida* infection, we studied the expression of above genes in *RIP2^−/−^* zebrafish larvae following *E. piscicida* challenge. As shown in Figures 8C,D, *RIP2^−/−^* zebrafish larvae infected with *E. piscicida* showed remarkable reduction for ENSDARG00000068621 (646.83-fold), MHC class I (2341.92-fold), mhc2dab (174.67-fold), and a modest, but significant reduction for ENSDARG00000088142 (2.20-fold), ENSDARG00000095200 (6.42-fold), ENSDARG00000090901 (6.53-fold), ENSDARG00000091499 (3.88-fold), ENSDARG00000089306 (3.13-fold), ENSDARG00000089179 (2.02-fold), ENSDARG00000079456 (3.68-fold), PA28 (4.18-fold), HSP70 (4.17-fold), β2M (8.66-fold), CD74a (3.16-fold), tapasin (4.26-fold), tapasin-like (3.56-fold), ctssb.2 (2.63-fold), mhc2 (1.86-fold), and mhc2β-like (10.18-fold). Taken together, these results show that RIP2 is involved in the regulation of NLRs signaling and MHC antigen presentation even in response to pathogen infection.

## Discussion

Although RIP2 has been cloned in zebrafish and goldfish (27, 28), the function of RIP2 was only limited for the activation of NF-κB signal pathway, the antibacterial and antiviral activities in RIP2 overexpressed cells *in vitro*. This study first demonstrated the signaling pathways regulated by piscine RIP2 in *RIP2^−/−^* zebrafish through transcriptome analysis. This study confirmed the crucial role of RIP2 in NLRs signaling and MHC antigen presentation, but not for MAPK and PI3K/Akt pathways.

NLRs, TLRs, and RLRs are three families of pathogen sensors, which have been suggested that there are extensive interplays among these PRRs (35). Initial studies using RIP2-deficient mice suggested that RIP2 was involved in TLR signaling (36). However, a later report showed that TLR signaling was intact in cells from RIP2-deficient mice, but RIP2 deficiency leads to abrogation of signaling in response to stimulation of NOD1 and NOD2 by their specific ligands or the intracellular pathogen *Listeria monocytogenes* (37), which indicated that RIP2 mediated NOD signaling, but not TLR signal transduction. Although piscine RIP2 deficiency significantly influenced “Toll-like receptor signaling pathway,” the target genes regulated by RIP2 were not TLRs or the key signaling molecules involved in TLR signal (Figure S2 in Supplementary Material). Different from TLRs, RIP2 deficiency significantly influenced the expression of other NLRs except for NOD1 and NOD2. Our previous studies revealed that multiple differentially expressed NLRs regulated by piscine NOD1 were mainly the homologs of NOD3/NLRC3 (30). This study also showed that piscine RIP2 was involved in the modulation of NLRs, especially the homologs of NOD3/NLRC3, which was identified as a negative regulator of innate immune signaling in mammals (38–40) and an essential negative regulator of macrophage activation and inflammation in teleost (41). Collectively, these findings suggest that the interplays between NOD1-RIP2 axis and other NLRs, such as NLRC3 signaling may contribute to the maintenance of immune homeostasis.

MHC class I and II pathways play essential roles in the activation of adaptive immune responses. Among NLRs, class II transactivator is a transcriptional coactivator that regulates IFNγ-activated transcription of MHC class I and II genes (42), whereas NLRC5 has been proven as MHC class I transactivator (43). Our previous report first identified NOD1 as a new regulator to drive the expression of MHC class I and II genes (30). Unexpectedly, RIP2 deficiency also significantly impaired the expression of those genes involved in MHC antigen presentation with or without pathogen infection, especially MHC class I and mhc2dab, a MHC class IIβ locus which contained similar conserved upstream regulatory sequences to human MHC class II genes (44). Our unpublished data showed that NOD1 can bind to the promoter of many MHC-related genes and induce their expression. Due to the lack of specific anti-RIP2 antibody to recognize the endogenous antigenic epitope at present, we were not able to validate the binding between RIP2 and MHC-related genes or other bridge molecules at DNA and protein levels. However, our preliminary results revealed that piscine RIP2 cooperated with histone H2 to induce the expression of MHC-related genes (unpublished data). Since RIP2 deficiency significantly impairs embryo and larval survival, the parents used for homozygotic propagation are too few. We will screen again the homozygotic *RIP2^−/−^* zebrafish to further confirm the correlation between RIP2 and histone H2.

In mammals, NOD1 and NOD2 activate gene transcription through the NF-κB and MAPK signaling pathways *via* the adaptor molecules RIP2 and CARD9 (45, 46). RIP2 is pivotal for NOD1- and NOD2-mediated NF-κB activation because NOD1 and NOD2 signaling is abolished in RIP2-deficient cells (12). Whereas, CARD9 has a critical function in NOD2-mediated activation of p38 and JNK MAPK pathways, but it was dispensable for NF-κB activation (46). In teleost, the transcriptional levels of NF-κB and MAPK pathways remained unchanged in NOD1-deficient zebrafish (30), however, NF-κB signaling pathway, but not MAPK pathway was significantly affected at the transcriptional level in RIP2-deficient zebrafish. This study also showed NOD1, but not RIP2 deficiency significantly impaired the protein levels of MAPK pathways. All these studies suggest that piscine NF-κB is activated in NOD1-independent, but RIP2-dependent manner; however, piscine MAPK pathways are activated *via* NOD1-dependent, but RIP2-independent manner.

In innate immunity, autophagy works downstream of PRRs, where it facilitates a number of effector responses, including the secretion of immune mediators, the control of inflammation, the control of adaptive immunity through the regulation of antigen presentation and the direct elimination of microorganisms (47). Cooperation between NLRs and autophagy in antimicrobial defense has been reported. NOD1 and NOD2 direct autophagy by recruiting the autophagy protein ATG16L1 to the plasma membrane at the bacterial entry site (48). NLRX1 and its interacting partner TUFM promote autophagy and IFN-I induction through associating with ATG5–ATG12 complexes and with ATG16L1 (49). Besides NLRs, RIP2 was also reported to be involved in autophagy. In NOD2-dependent autophagy, RIP2 tyrosine kinase activity plays a dual role by sending a positive autophagy signal through activation of p38 MAPK and relieving repression of autophagy mediated by the phosphatase PP2A (50). In early endosomes, NOD1 interacts with peptidoglycan and RIP2, and promotes RIP2-dependent autophagy and inflammatory signaling in response to bacterial infection (21). RIP2 also regulated mitophagy in a kinase-dependent manner by phosphorylating the mitophagy inducer ULK1 during viral infection (51). In zebrafish, autophagy is active in early embryonic development. Autophagy gene knockdown resulted in developmental defects and reduced organismal survival (32). Our results provide the first evidence that NOD1-RIP2 axis contribute to larval survival and the modulation of autophagy-associated proteins during vertebrate normal development. The exact mechanisms involved in NOD1-RIP2 axis mediated autophagy will be further addressed in future experiments.

CD44 is a major cell surface receptor which serves to trigger and direct the cellular response to hyaluronan (52). It has been recognized that CD44 also functions as a signaling receptor in a variety of cell types, which physically associates with intracellular protein tyrosine kinase Lck and Fyn (53). Recent studies have identified a cross talk between CD44 and TLRs or NLRs. CD44 is important in providing a brake for innate immune inflammatory responses by promoting the expression of negative regulators of TLR4 signaling (54) or negatively regulating *in vivo* inflammation mediated by TLRs *via* NF-κB activation (55). In addition, CD44 is also critical for cell survival of various cell types through activating PI3K-Akt signaling pathway (56, 57). In teleost, we found CD44a was required for NOD1-mediated PI3K-Akt pathway and larval survival (30). Interesting, although RIP2 deficiency significantly impaired the expression of CD44a, the downstream signaling of Lck-PI3K-Akt pathway remained unchanged. All these findings suggest that NOD1-mediated CD44a-Lck-PI3K-Akt pathway is independent of RIP2 (Figure 8E). Since CD44a is reported to be a multifunction protein, such as mediating T-cell extravasation and regulating T-cell development (58, 59), the biological significances of expression regulation of CD44a-mediated by RIP2 need to be further studied.

In conclusion, this study revealed the modulation of immune signaling pathways by RIP2 during zebrafish larval development. Based on the experimental data mentioned above and the previous reports, a working model of how piscine NOD1-RIP2 axis affects larvae survival and intracellular immune signaling is shown in Figure 8E. NOD1-RIP2 axis contributes to the modulation of NLRs signaling, MHC antigen presentation, and autophagy. Different from this, MAPK pathways and the downstream signaling of CD44a-Lck-PI3K-Akt mediated by NOD1 is independent of RIP2 (Figure 8E). Future studies, including the interplays and corresponding mechanisms between NOD1-RIP2 axis and NLRC3 signaling, the correlation between RIP2 and histone H2 in the regulation of MHC-related genes, the exact mechanisms involved in NOD1-RIP2 axis mediated autophagy, and the biological significances of expression regulation of CD44a mediated by RIP2, will provide further insight into the molecular signaling mechanisms of NOD1-RIP2 axis in the maintenance of immune homeostasis which is benefic to larval survival.

## Ethics Statement

All animal experiments were conducted in accordance with the Guiding Principles for the Care and Use of Laboratory Animals and were approved by the Institute of Hydrobiology, Chinese Academy of Sciences (Approval ID: IHB 2013724).

## Author Contributions

MC conceived and designed the experiments. MC and WC analyzed the data. MC, XW, YH, and LC performed the experiments. MC wrote and revised the manuscript. PN revised the manuscript. All authors reviewed the manuscript.

## Conflict of Interest Statement

The authors declare that the research was conducted in the absence of any commercial or financial relationships that could be construed as a potential conflict of interest.
