# Supplementary material for: RIP2 Is a Critical Regulator for NLRs Signaling and MHC Antigen Presentation but Not for MAPK and PI3K/Akt Pathways
Source: Front Immunol. 2018 Apr 10;9:726. doi: 10.3389/fimmu.2018.00726 (PMC5903030; doi:10.3389/fimmu.2018.00726)
Supplement: Supplementary file 1 [file Data_Sheet_1.DOCX]

Figure S1


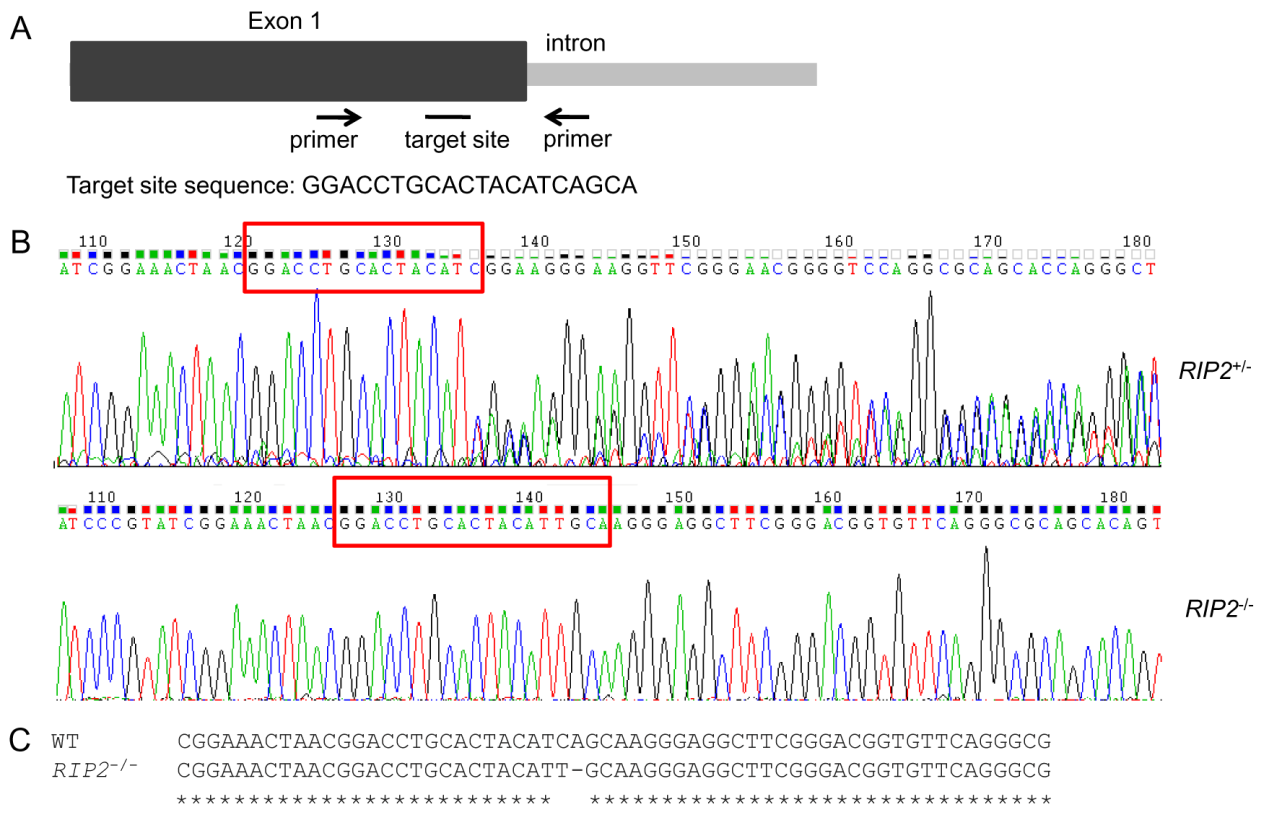


Figure S1: Cas9/gRNA induces indels in the RIP2 locus in zebrafish. (A) Cartoon showing the position of the target site and its sequence in the zebrafish RIP2 locus. (B) Sequencing results of PCR amplicons from the heterozygous and homozygous zebrafish in the targeted RIP2 locus. (C) Sequence alignment of the targeted RIP2 locus in WT and RIP2^-/-^ zebrafish.

Figure S2


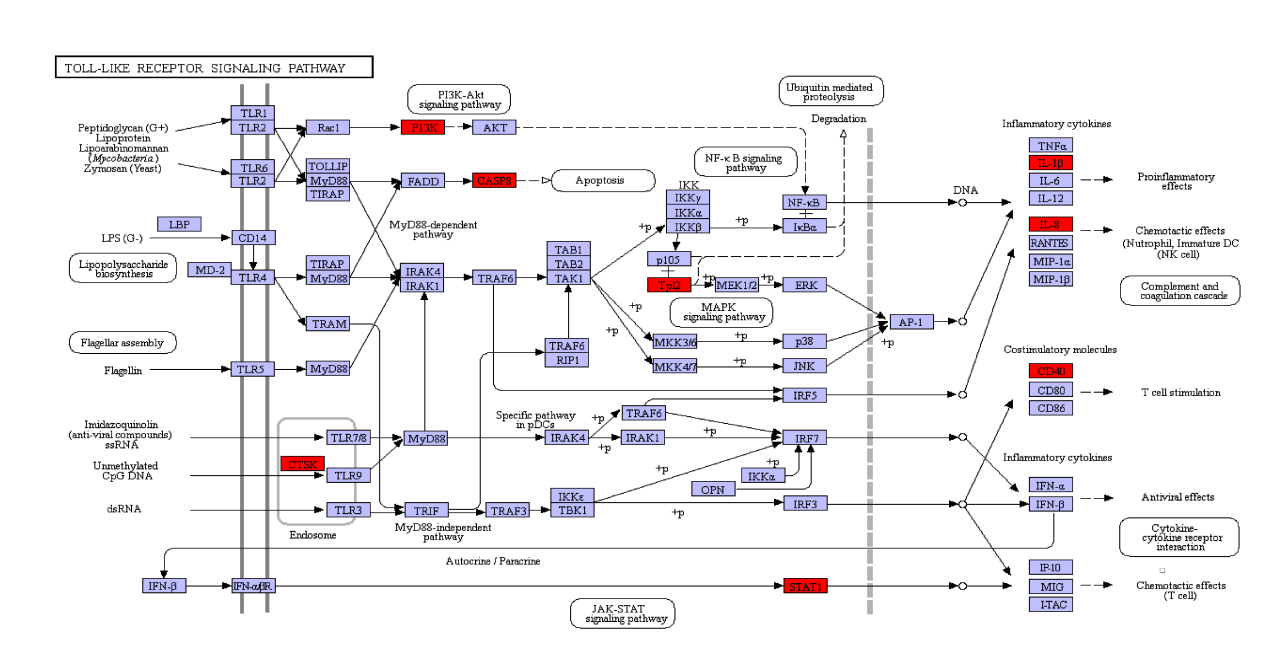


Figure S2: TLR signal regulated by RIP2 deficiency. Differentially expressed genes (DEGs) were shown in red.
